# Infantile Mastocytoma: Clinical Presentation and Diagnostic Insights

**DOI:** 10.7759/cureus.108107

**Published:** 2026-05-01

**Authors:** Khedija Bennani, Salim Gallouj, Meryem El Bakkali, Ouiame El Jouari

**Affiliations:** 1 Dermatology, Abdelmalek Essaadi University, Faculty of Medicine and Pharmacy, University Hospital Mohammed VI, Tangier, MAR

**Keywords:** cutaneous mastocytosis, histopathology, infantile mastocytoma, mast cells, pediatric dermatology

## Abstract

Mastocytosis is a heterogeneous group of disorders characterized by abnormal proliferation and accumulation of mast cells in one or more organs. Cutaneous mastocytosis (CM) is the most common form in children, with solitary mastocytoma representing a distinct subtype. Mastocytomas are generally benign, present at birth or in early infancy, and may manifest as pigmented macules, nodules, or plaques, occasionally associated with flushing or urtication. Histopathological confirmation is essential in atypical presentations.

We report a 4.5-month-old infant presenting with a lesion on the metacarpophalangeal joint of the right hand, first noted at birth. The lesion initially appeared erythematous, then developed as bullous eruptions with serous fluid, and eventually evolved into a reddish, crusted nodule with a rough surface. The infant experienced intermittent, brief episodes of generalized flushing but had no diarrhea, systemic symptoms, or developmental delay. Physical examination was otherwise unremarkable, and laboratory tests, including complete blood count, liver and renal function, and serum tryptase, were within normal limits. A skin biopsy revealed a dense dermal infiltrate of monomorphic mast cells with round to oval nuclei and abundant eosinophilic granules. Toluidine blue staining confirmed metachromatic granules, consistent with a solitary mastocytoma.

Mastocytomas typically present as solitary reddish-brown nodules or plaques, often exhibiting Darier’s sign, although this may be absent or unsafe to elicit. Our case demonstrates an uncommon bullous transformation preceding the classic nodular appearance. Episodic flushing suggested mast cell mediator release; however, the absence of systemic involvement and normal tryptase levels confirmed a purely cutaneous form. Histological examination with toluidine blue or immunohistochemical stains (CD117, tryptase) remains the diagnostic gold standard. Management is usually conservative, with antihistamines for symptomatic relief. Most lesions regress spontaneously by two to three years of age, and prognosis is excellent in the absence of systemic disease.

This case highlights an under-recognized presentation of infantile mastocytoma, emphasizing the importance of clinical awareness and histopathological confirmation. Accurate diagnosis allows appropriate reassurance for caregivers, while long-term monitoring ensures early detection of any rare progression to systemic mastocytosis.

## Introduction

Mastocytosis comprises a heterogeneous group of disorders involving abnormal growth and accumulation of mast cells in one or more organ systems. Mast cells are immune cells that play a key role in inflammatory and allergic responses through the release of mediators such as histamine, which can lead to symptoms including flushing, pruritus, and urtication. Mastocytosis is generally classified into cutaneous and systemic forms, with cutaneous mastocytosis (CM) being more common in pediatric populations. Among the various forms of CM, solitary mastocytoma is one of the rarest presentations in infants and children [1].

Mastocytomas are usually benign and self-limiting, often presenting within the first few months of life. Their clinical manifestations may include pigmented macules, nodules, or plaques, occasionally associated with urtication or flushing. However, atypical presentations may occur and can pose diagnostic challenges, particularly when clinical features are not characteristic. Diagnosis is mainly based on clinical suspicion, supported by histological and cytochemical findings [2]. Herein, we report a case of nodular mastocytosis in an infant, emphasizing the importance of histological confirmation in atypical presentations.

## Case presentation

**Figure 1:**
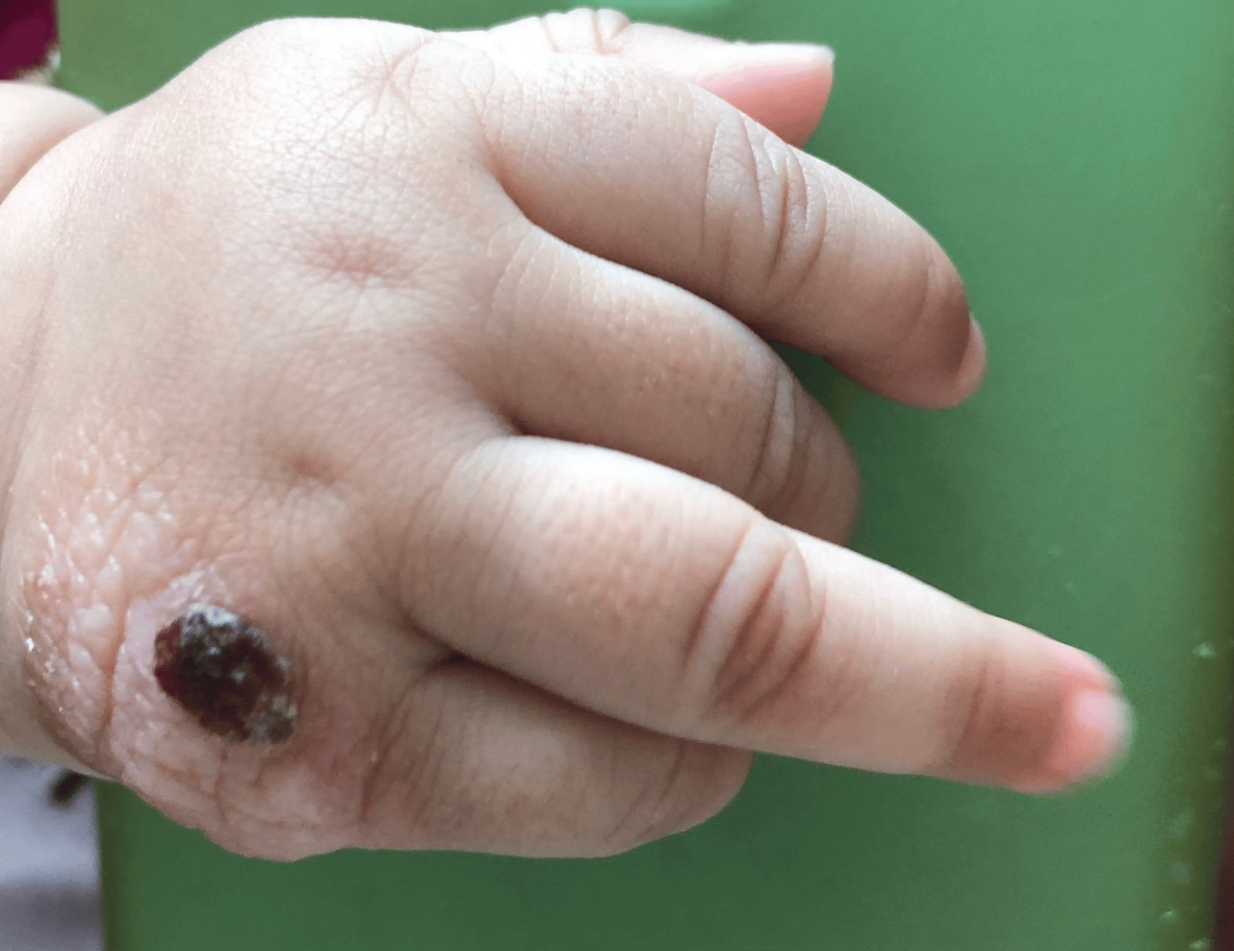
Clinical presentation A solitary reddish-brown nodule is observed on the metacarpophalangeal joint of the right hand. The lesion has a rough, crusted surface with irregular borders. No surrounding erythema or signs of infection are noted. The nodule corresponds to the site of previous bullous eruptions and represents the typical presentation of a mastocytoma in infancy.

A 4.5-month-old infant was brought in with a skin lesion on the metacarpophalangeal joint of the right hand, first noticed at birth. Initially erythematous, the lesion progressed to small bullous eruptions containing clear fluid. Over time, it evolved into a reddish, crusted nodule with a rough surface (Figure 1). The lesion was solitary, and Darier’s sign was not assessed due to the bullous and fragile nature of the lesion, in order to avoid potential local exacerbation.

The infant also experienced intermittent episodes of generalized flushing, each lasting less than two minutes. There were no associated gastrointestinal symptoms, systemic complaints, or developmental delays. The child’s overall condition remained stable, and physical examination revealed no hepatosplenomegaly or lymphadenopathy. Darier’s sign was not tested to avoid aggravating the lesion.

**Figure 2:**
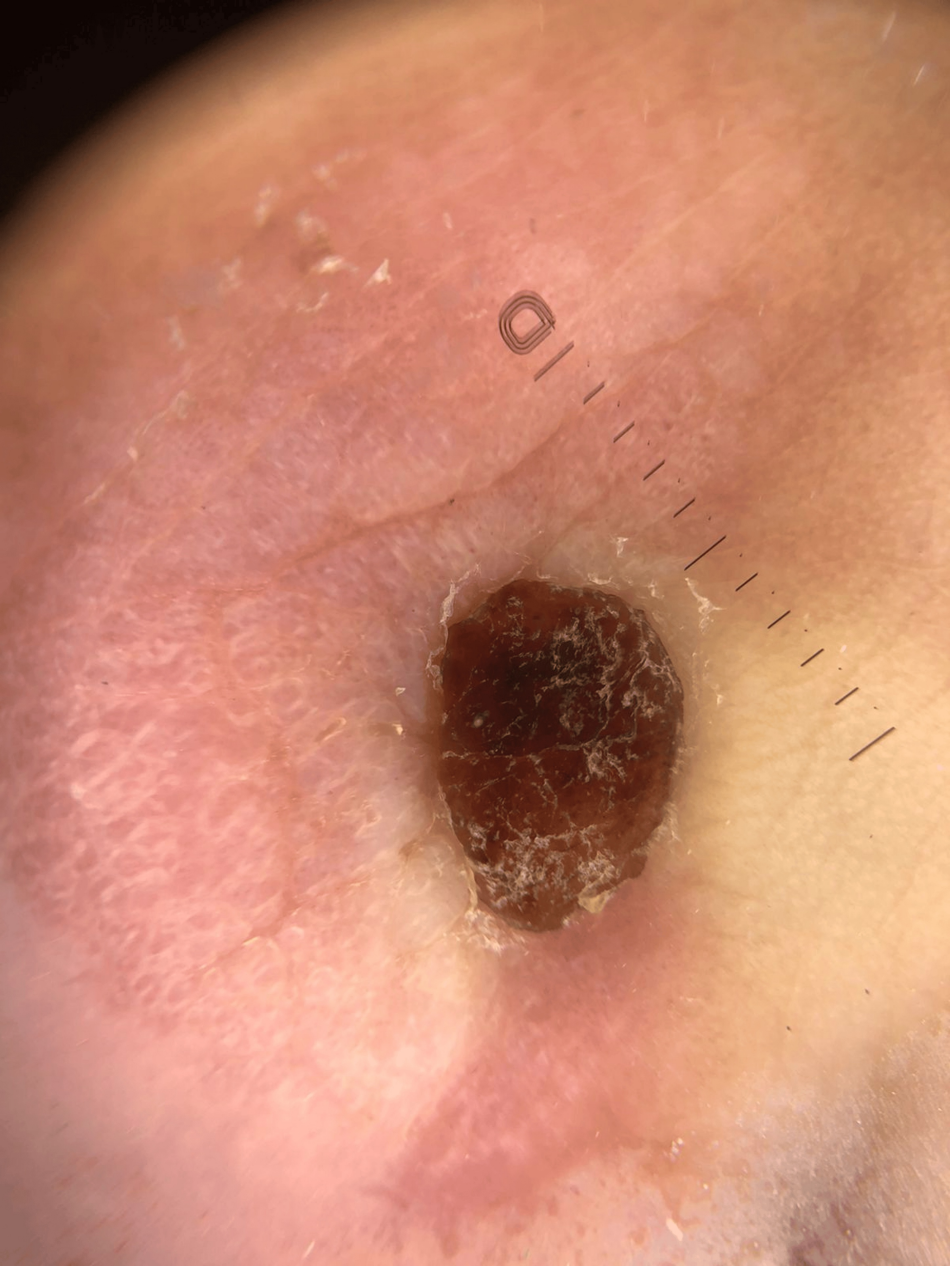
Dermoscopy of the lesion Dermoscopy reveals a homogeneous, light-brown to reddish structure with a slightly nodular surface. No significant vascular structures or pigment network are observed.

Dermoscopy of the lesion showed a homogeneous light-brown to reddish structure with a slightly nodular surface (Figure 2). No significant vascular patterns or pigment network were observed, consistent with a solitary cutaneous mastocytoma.

Laboratory evaluations, including complete blood count, liver and kidney function tests, and serum tryptase levels, were all within normal limits (Table 1).

**Table 1:** Laboratory findings

Test	Patient value	Reference range (infant)	Interpretation
Hemoglobin	13.5 g/dl	11.0-14.5 g/dl	Normal
White blood cells	9.0×10³/μL	6-17×10³/μL	Normal
Platelets	280×10³/μL	150-450×10³/μL	Normal
Aspartate aminotransferase (serum glutamic-oxaloacetic transaminase)	35 U/L	20-60 U/L	Normal
Alanine aminotransferase (serum glutamic-pyruvic transaminase)	30 U /L	7-56 U/L	Normal
Creatinine	0.3 mg/dL	0.2-0.5 mg/dL	Normal
Blood urea nitrogen	8 mg/dL	5-18 mg/dL	Normal
Tryptase	3 μg/L	<11.4 μg/L	Normal

**Figure 3:**
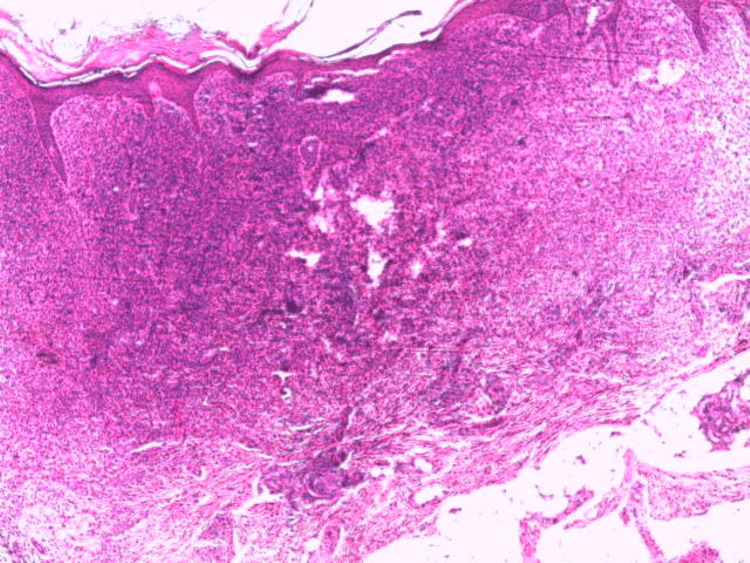
Histological findings (H&E, ×50) Dense dermal infiltrate of monomorphic mast cells with round to oval nuclei and granular cytoplasm, extending into the hypodermal compartments. H&E: hematoxylin and eosin.

**Figure 4:**
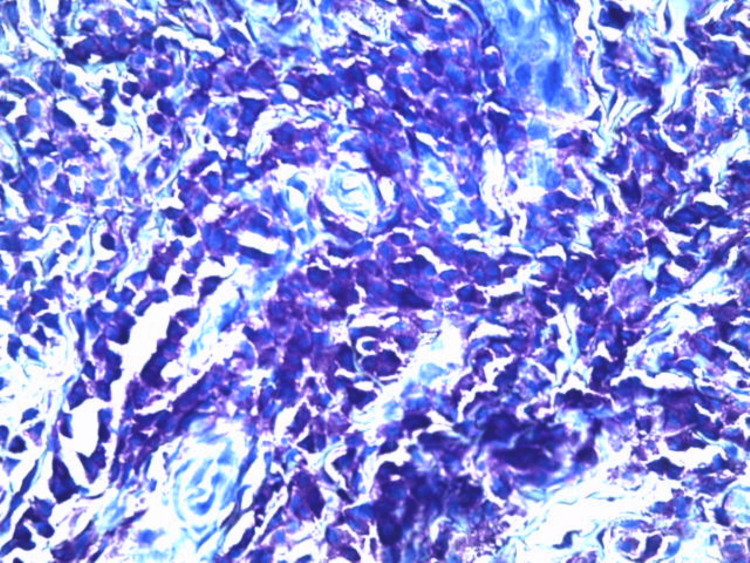
Toluidine blue, ×400 Mast cells exhibit abundant cytoplasmic granules showing metachromatic staining, characteristic of mast cells. The granules are densely packed and prominently highlight the mast cell population within the dermis.

A skin biopsy demonstrated a dense dermal infiltrate composed predominantly of large, monomorphic mast cells (Figure 3), with round-to-oval nuclei and abundant cytoplasm containing eosinophilic granules (Figure 4). Toluidine blue staining confirmed the presence of metachromatic granules, consistent with mast cell infiltration. The histological features were diagnostic of solitary mastocytoma.

Based on the combination of clinical presentation and histopathological findings, a diagnosis of cutaneous mastocytoma was established. The patient was managed conservatively and remained stable during follow-up, with no new lesions or systemic symptoms reported.

## Discussion

Solitary mastocytoma represents the most frequent form of cutaneous mastocytosis in pediatric patients, despite being uncommon in absolute terms. It typically presents as a solitary, reddish-brown nodule or plaque, often evident at birth or within the first few months of life [2,3]. These lesions can exhibit urtication upon mechanical stimulation (Darier’s sign) due to mast cell degranulation, although this reaction is not always present and may be unsafe to elicit in fragile lesions [3,4]. Clinically, most mastocytomas are benign and self-limiting, with spontaneous regression usually occurring by two to three years of age [5].

Our case demonstrates a less typical course, with an initial bullous transformation preceding the classic nodular appearance. Such presentations are uncommon but highlight the spectrum of clinical manifestations of infantile mastocytomas [2,6]. The intermittent flushing episodes observed in our patient suggest transient systemic release of mast cell mediators. However, normal serum tryptase levels and the absence of systemic involvement support a purely cutaneous form, consistent with previously described pediatric cases [6].

Histopathological examination remains the gold standard for diagnosis, particularly in atypical or diagnostically uncertain cases [4]. Mast cell identification can be reliably achieved using toluidine blue or Giemsa staining, which reveal metachromatic cytoplasmic granules, or through immunohistochemistry with markers such as CD117 and tryptase [7]. In our patient, both hematoxylin and eosin staining (HES) and toluidine blue staining confirmed a dense dermal mast cell infiltrate extending into the hypodermis, consistent with solitary mastocytoma. Dermoscopy can provide additional non-invasive diagnostic clues, although it does not replace histological confirmation [2].

Management of mastocytomas is generally conservative. Symptomatic relief, such as controlling pruritus or flushing, can be achieved with oral antihistamines [5]. High-potency topical corticosteroids may also be used in selected symptomatic cases. Surgical excision is rarely indicated, except in cases of persistent, symptomatic, or cosmetically concerning lesions. Long-term prognosis in pediatric mastocytomas is excellent, with most lesions resolving spontaneously without progression to systemic mastocytosis [5]. Nevertheless, careful follow-up is recommended, as rare cases may evolve into systemic disease or present with atypical clinical features [1,4].

This case emphasizes the importance of recognizing the variable clinical and histopathological spectrum of infantile mastocytomas. Early and accurate diagnosis allows appropriate counseling for caregivers, prevents unnecessary interventions, and ensures vigilance for the rare possibility of systemic involvement. Awareness of less common manifestations, including bullous changes and episodic flushing, may facilitate early diagnosis and appropriate management in pediatric patients [2,6,7].

## Conclusions

This case highlights an uncommon presentation of infantile mastocytoma, with bullous transformation preceding the classic nodular lesion and episodic flushing. Recognition of such atypical features, combined with histopathological confirmation, is essential for accurate diagnosis and appropriate management. Mastocytomas are generally benign and self-limiting, with an excellent prognosis in the absence of systemic involvement. Careful follow-up remains important to monitor for any rare progression or atypical evolution. Early diagnosis and parental counseling can prevent unnecessary interventions and provide reassurance to caregivers.
